# Point Cloud Registration Method Based on Geometric Constraint and Transformation Evaluation

**DOI:** 10.3390/s24061853

**Published:** 2024-03-14

**Authors:** Chuanli Kang, Chongming Geng, Zitao Lin, Sai Zhang, Siyao Zhang, Shiwei Wang

**Affiliations:** 1 College of Geomatics and Geoinformation, Guilin University of Technology, Guilin 541004, China; 2014012@glut.edu.cn (C.K.); 2120211878@glut.edu.cn (Z.L.); 1020211817@glut.edu.cn (S.Z.); 2120211915@glut.edu.cn (S.Z.); 2120222025@glut.edu.cn (S.W.); 2 Key Laboratory of Spatial Information and Geomatics, Guilin University of Technology, Guilin 541004, China

**Keywords:** geometric constraint, point cloud registration, transformation estimation, evaluation of registration

## Abstract

Existing point-to-point registration methods often suffer from inaccuracies caused by erroneous matches and noisy correspondences, leading to significant decreases in registration accuracy and efficiency. To address these challenges, this paper presents a new coarse registration method based on a geometric constraint and a matrix evaluation. Compared to traditional registration methods that require a minimum of three correspondences to complete the registration, the proposed method only requires two correspondences to generate a transformation matrix. Additionally, by using geometric constraints to select out high-quality correspondences and evaluating the matrix, we greatly increase the likelihood of finding the optimal result. In the proposed method, we first employ a combination of descriptors and keypoint detection techniques to generate initial correspondences. Next, we utilize the nearest neighbor similarity ratio (NNSR) to select high-quality correspondences. Subsequently, we evaluate the quality of these correspondences using rigidity constraints and salient points’ distance constraints, favoring higher-scoring correspondences. For each selected correspondence pair, we compute the rotation and translation matrix based on their centroids and local reference frames. With the transformation matrices of the source and target point clouds known, we deduce the transformation matrix of the source point cloud in reverse. To identify the best-transformed point cloud, we propose an evaluation method based on the overlap ratio and inliers points. Through parameter experiments, we investigate the performance of the proposed method under various parameter settings. By conducting comparative experiments, we verified that the proposed method’s geometric constraints, evaluation methods, and transformation matrix computation consistently outperformed other methods in terms of root mean square error (RMSE) values. Additionally, we validated that our chosen combination for generating initial correspondences outperforms other descriptor and keypoint detection combinations in terms of the registration result accuracy. Furthermore, we compared our method with several feature-matching registration methods, and the results demonstrate the superior accuracy of our approach. Ultimately, by testing the proposed method on various types of point cloud datasets, we convincingly established its effectiveness. Based on the evaluation and selection of correspondences and the registration result’s quality, our proposed method offers a solution with fewer iterations and higher accuracy.

## 1. Introduction

Laser sensors utilize the principle of laser ranging to record the three-dimensional coordinates, reflectivity, and texture information from the object being scanned. In practical applications, data are typically acquired from different angles due to limitations imposed by lines of sight, measurement methods, and the geometry of the objects that are being scanned. Each point cloud has its own local reference frames, and the goal of point cloud registration is to unify the multiple angle point cloud data into a common coordinate system [1].

Point cloud registration can be divided into two methods: coarse registration and fine registration [2]. In the context of fine registration methods, the iterative closest point (ICP) algorithm [3] is most commonly used. This algorithm utilizes the least squares method to compute the matching point sets and achieves convergence through iterative optimization, resulting in satisfactory matching results. There were also variants of ICP. The point-to-plane ICP registration algorithm proposed by Low, Kok-Lim et al. [4] demonstrated that using the point-to-plane approach to calculate the transformation matrix is faster and achieves higher registration accuracy compared to the point-to-point approach. S. Rusinkiewicz [5] proposed a symmetric version of the iterative point-to-plane ICP algorithm, which also introduces an alternative method called rotation linearization to simplify the optimization process into a linear least squares problem. ICP is characterized by its straightforward approach and showed its effectiveness, particularly when registering data with a solid initial alignment [6]. Therefore, the challenge of obtaining accurate initial correspondences still persists.

A coarse registration method based on the selection of matching point pairs can be implemented through five steps: keypoint detection, local feature description of keypoints, correspondences selecting, and computation of the optimal transformation matrix [7]. Keypoint detection aims to identify a small set of distinct points from a point cloud to expedite registration, as raw point clouds often contain a large number of points. Local feature description involves using rotation-invariant feature vectors to capture geometric and spatial information within a local surface. By comparing these local geometric features using distance metrics, point-to-point correspondences can be established. However, there are still significant outliers in the initial correspondences. This is due to issues such as keypoint localization, the challenge of distinguishing repeatable patterns using local geometric features, and limited overlap between the point cloud views being aligned. Some of these methods are proposed in order to select out high-quality correspondences [8]. For example, Mian et al. proposed a similarity score based on point cloud descriptors [9]. This method determines correspondences based on the differences between feature elements in point cloud descriptors. However, issues such as data noise, occluded regions, and repeated features, can lead to misjudgment, so this method can only serve as a baseline for evaluation. Another approach is to select high-quality correspondences based on geometric constraints between correspondences. For instance, a base line algorithm is Lowe’s ratio rule [10], which determines whether to accept a pair of matching points based on the ratio of the nearest distance to the second-nearest distance. A significant distance difference indicates that the point has better discriminability in the feature space. H. Chen et al. [11] proposed geometric consistency, which imposes constraints based on the geometric distance differences between the source and target points of the two matching points. This approach aims to select correspondences that meet a threshold. However, these methods mostly only utilize a single constraint, leaving room for improvement in the quality of matching point pairs. Moreover, there is the potential of finding the optimal result based on these constraints.

In the field of 3D point cloud registration, there are various algorithms available to solve the outliers’ issue. At present, many coarse registration methods were proposed. For example, the compatibility-guided sampling consensus (GC-SAC) registration method proposed by S. Quan and J. Yang [12] utilizes rigidity constraints and salient points’ distance constraints for correspondences selection, and determines the optimal transformation matrix based on the maximum number of inliers. Guo et al. [13] proposed a method for point cloud registration using rotation projection statistics (RoPS) features for correspondences feature matching, followed by transformation estimation methods and the ICP algorithm. However, this method relies solely on the initial correspondences generated from descriptors and does not eliminate low-quality correspondences. Another method, proposed by Yang et al. [14], is the consistency voting method, which ranks the correspondences based on constraints such as rigidity and local reference frames. This approach provides a means to prioritize and assess correspondences for improved accuracy and efficiency. Buch et al. [8] proposed a method for confirming inlier points by using Lowe’s ratio and the minimum ratio of geometric distances between target points and source points. Sun et al. [15] presented a solution called inlier searching using compatible structures (ICOS), which constructs a compatible structure to facilitate subsequent outlier removal and inlier searching. They designed three efficient frameworks for estimating rotation matrices, known-scale registration, and unknown-scale registration. Rodolà et al. [16] proposed a sparse point matching approach based on game theory; Tombari et al. [17] introduced a method called 3D Hough voting (3DHV), which involves voting based on the positional information of correspondences in the Hough space; Sahloul et al. [18] presented a two-stage voting scheme that uses dense evaluation and ranking of local and global geometric consistency to distinguish inliers. Quan, S. et al. [19] proposed a robust method, progressive consistency voting (PCV), for feature matching in 3D point clouds. It assigns confidence scores to correspondences based on geometric consistency and utilizes a voting-based scheme. Xu, G. et al. [20] proposed a method that combines RANSAC, intrinsic shape signatures (ISS), and 3D shape context descriptor (3DSC) to improve the ICP registration of large point clouds. It uses voxel grid filter for down-sampling, extracts keypoints with ISS, describes them with 3DSC, performs coarse registration with RANSAC using ISS-3DSC features, and achieves accurate registration with ICP. In the subsequent experiments, we will refer to this method as IRIS (improved registration using ISS, RANSAC, and ICP with 3DSC). Yan, L. et al. [21] proposes a graph reliability outlier removal (GROR) method, which is a strategy based on the reliability of the correspondence graph to address the issue of outliers in point cloud registration.

Some methods utilize other characteristics of point clouds for registration, Liang, L. et al. [22] introduce an innovative affine iterative closest point algorithm incorporating color information and correntropy. By integrating color features into traditional affine algorithms, the method established more accurate and reliable correspondences. Liu, J et al. [23] proposed point cloud registration with multilayer perceptrons (PCRMLP), a novel model for urban scene point cloud registration that achieves comparable registration performance to prior learning-based methods. PCRMLP estimates transformations implicitly from concrete instances using semantic segmentation and density-based spatial clustering of applications with noise (DBSCAN) to generate instance descriptors, enabling robust feature extraction, dynamic object filtering, and logical transformation estimation.

In the context of computing the optimal transformation matrix, a commonly used method is the random sample consensus (RANSAC) algorithm proposed by Fischler et al. [24]. This algorithm randomly selects three correspondences from the point sets, generates a rotation and translation matrix, and then counts the inlier points that are within a distance threshold under this transformation matrix. This step is repeated multiple times, and the transformation matrix with the maximum number of inlier points is selected as the final result, while outlier points are discarded. However, the RANSAC method still necessitates a minimum of three correspondences for generating a transformation matrix, and the randomness in selecting these correspondences introduces inaccuracy to its registration result. In contrast, our proposed registration algorithm based on two-point correspondences enhances the precision of the final registration result since these correspondences are filtered through geometric constraints. This approach also saves time compared to the RANSAC, which involves traversing to generate various transformation matrices. Additionally, the sampling process only selects transformation matrix results based on a single criterion, such as a distance threshold, which can potentially miss the optimal transformation matrix. Quan et al. [25] extended the RANSAC algorithm by establishing local reference frames on key points, enabling the inference of transformation matrices with only one correspondence. They also proposed a “maximum overlapping point set” criterion to evaluate the registration results. Another improved variant of the RANSAC algorithm is the optimized sample consensus (OASC) algorithm proposed by Yang et al. [26], which introduces a new error metric. However, these methods only employ a single evaluation criterion for assessing the registration results. One of the research directions of this paper is to explore whether combining multiple evaluation factors would yield improved registration results. By considering various evaluation factors simultaneously, such as alignment accuracy, robustness to outliers, and computational efficiency, it is possible to gain a more comprehensive understanding of the registration performance.

**Figure 1:**
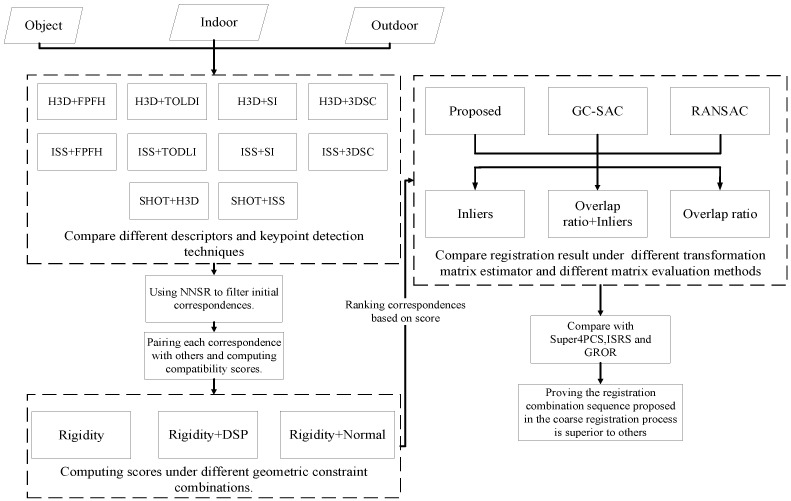
The architecture of the experiment in our article.

To address the aforementioned issues, this paper proposes a method based on the rigidity and salient points’ distance constraints to select out two-by-two correspondences that fit the standard. Then, based on the centroid of two points, we can calculate their translation matrix. Based on the vector of the two points and their salient points, we can construct their two-point rotation pose (reference frame). By combining the two matrices mentioned earlier, we obtain a transformation matrix. With the transformation matrix of the source and target point clouds known, we obtain the transformation matrix of the source point cloud by reverse deduction. Finally, we proposed a transformation evaluation method combined with overlap ratio and inlier points to select the best transformation matrix. The overall experimental architecture of the paper is shown in Figure 1. In summary, the main contributions of this paper are:

(1) We propose an evaluation system based on rigidity constraints and distance of salient point constraints to filter out outliers in the initial correspondences. The rigidity constraint focuses on the geometric relationship between points, while the DSP constraint leverages the context information within the local surface. By combining them, we can better select the top N candidates from the correspondences.
(2) We propose a method to compute the transformation matrix of the source point cloud in reverse by computing the rotation and translation matrices between the source and target points based on the correspondences. Considering two high-quality correspondences from the samples for calculating the transformation matrix allows us to generate matrix with few iterations and also increases the probability of finding the optimal matrix.
(3) To further refine the registration process, we propose an evaluation method based on the overlap ratio and inlier points. This evaluation method allows us to search for the best registration result among the top N candidates in a more comprehensive manner.

## 2. The Principle of Proposed Registration

**Figure 2:**
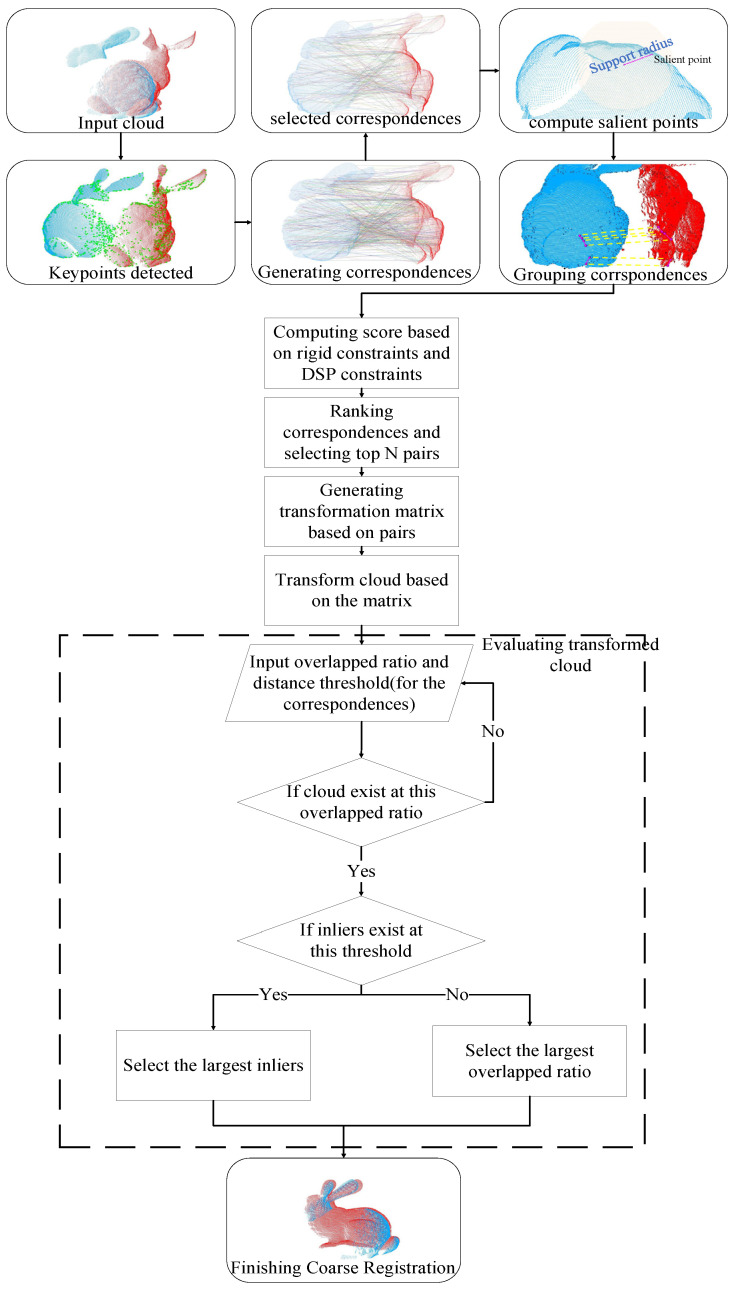
The workflow of the coarse point cloud registration method based on geometric constraints and the two-factors evaluation. The red object represents the target point cloud and the blue one represents the source point cloud. The green points represents keypoins that are detected from the object. In Grouping correspondences, the yellow dotted line represents the correspondences, and the purple line represents the combination of two correspondences.

First, this paper adopts a keypoint detection method and feature descriptor to generate initial correspondences. Then, we apply the nearest neighbor similarity ratio to filter these initial correspondences and select higher quality ones. Next, within each correspondence, we compute salient points for the source and target points, and combine them pairwise. Based on rigidity and salient points’ distance constraint, we select higher quality ones. Next, within each point pair, we compute salient points for the source and target points, and combine them pairwise. Based on rigidity and salient points’ distance constraint, we select out correspondences with higher scores. Finally, using the local reference frames between the two points and their centroid positions, we compute the transformation matrix. The best matrix is selected based on the overlap ratio and the number of inlier points. The technical flow of the entire method is shown in Figure 2.

### 2.1. Generate Correspondences

First, keypoints are extracted from the source and target point clouds, using keypoints extraction methods such as Harris 3D (H3D) [27] and intrinsic shape signatures (ISS) [28]. Then we combine them with descriptors such as fast point feature histogram (FPFH) [29], 3D shape context descriptor (3DSC) [30], signature of histograms of orientations (SHOT) [31], and spin image (SI) [32] for comparative experiments. These descriptors are used to generate initial correspondences using the similarity score algorithm [6]. Based on the comparative results mentioned in the subsequent experiments, we choose to use the combination of Harris 3D keypoint detection and the local image descriptors obtained from the triplet orthogonal views based on the newly proposed local reference frame (LRF), known as triple orthogonal local depth images (TODLI) [33], for generating initial correspondences.

After obtaining the keypoints using Harris 3D, we generate histograms based on the TOLDI descriptors for both the keypoints in the source point cloud, denoted as $p^{s}$ , and the keypoints in the target point cloud, denoted as $p^{t}$ . We calculate the correspondences’ differences between the feature values of the histograms and select the keypoint pairs with the smallest differences as the initial correspondences, as defined in (1).

(1) $$c=\arg\min{\left\|f^{s}-f^{t}\right\|}_{L_{2}}$$

In this step, we calculate the feature distance between feature $f^{s}$ in the source point cloud and feature $f^{t}$ in the target point cloud. We select the correspondence c with the smallest feature distance as the initial correspondences. Next, we use the NNSR [10] to improve the quality of the correspondences. The NNSR is defined as the ratio of the feature distances between the source point cloud and the target point cloud in each correspondence, as shown in (2). All correspondences are sorted based on this ratio. The ratio can be used to measure the uniqueness and accuracy of the correspondences. Generally, correspondences with a larger feature distance ratio (greater difference between the closest and second closest distances) tend to have higher uniqueness and are assigned higher scores. By applying the NNSR filter, we can further reduce the influence of incorrect matches and noise point pairs, and select higher quality correspondences as the final set of initial correspondences.

(2) $$s_{\mathrm{Ratio}}\;(c)=1-\frac{d\left(f^{s},f_{1}^{t}\right)}{d\left(f^{s},f_{2}^{t}\right)}$$

The features $f_{1}^{s}$ and $f_{2}^{t}$ represent the closest and second closest features, respectively, to the feature $f^{s}$ in the source point cloud $p^{s}$ . In practical applications, the choice of the appropriate number of correspondences can be based on specific requirements and tasks. Selecting an appropriate number of point pairs helps balance the registration accuracy, computational complexity, and robustness. We will conduct this experiment in the following part to find the appropriate parameter.

### 2.2. Selecting Correspondences Based on Geometric Constraint

By incorporating various geometric constraints, additional information and constraints can be provided in the point cloud registration process, assisting in the selection of matching point pairs and further enhancing their quality. In this section, we introduce three constraint methods that primarily rely on these two correspondences, $c_{1}$ and $c_{2}$ .

**Figure 3:**
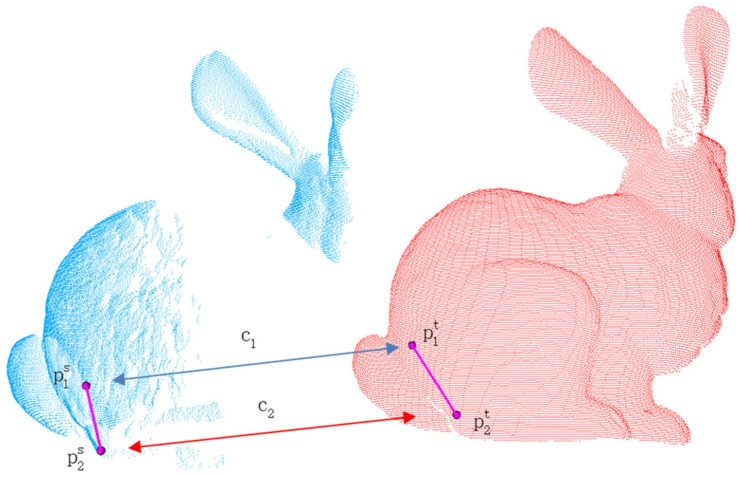
Illustration of a rigidity constraint, consisting of any two correspondences. The red bunny represents the target point cloud and the blue one represents the source point cloud.

The rigidity constraint [8] is based on the invariance property and determines whether the two points from the source point cloud and the two points from the target point cloud should be considered as the same correspondence based on the difference in their distances. This constraint is represented by (3) and illustrated in Figure 3.

(3) $$S_{\mathrm{rigidity}}=\left|d\left(p_{1}^{s},p_{2}^{s}\right)-d\left(p_{1}^{t},p_{2}^{t}\right)\right|$$

The distributions of $d(p_{1}^{s},p_{2}^{s})$ and $d(p_{1}^{t},p_{2}^{t})$ represent the Euclidean distances between the points $p_{1}^{s}$ and $p_{2}^{s}$ within the source correspondences, as well as between the points $p_{1}^{t}$ and $p_{2}^{t}$ within the target correspondences. In the process of point cloud registration, the rigidity constraint serves as a fundamental constraint that provides an initial estimation for subsequent optimization and refinement steps. However, the estimation based on the rigidity constraint is typically coarse and can lead to multiple rigid transformations that satisfy the constraint. Therefore, it is necessary to combine other constraints to reduce ambiguity.

**Figure 4:**
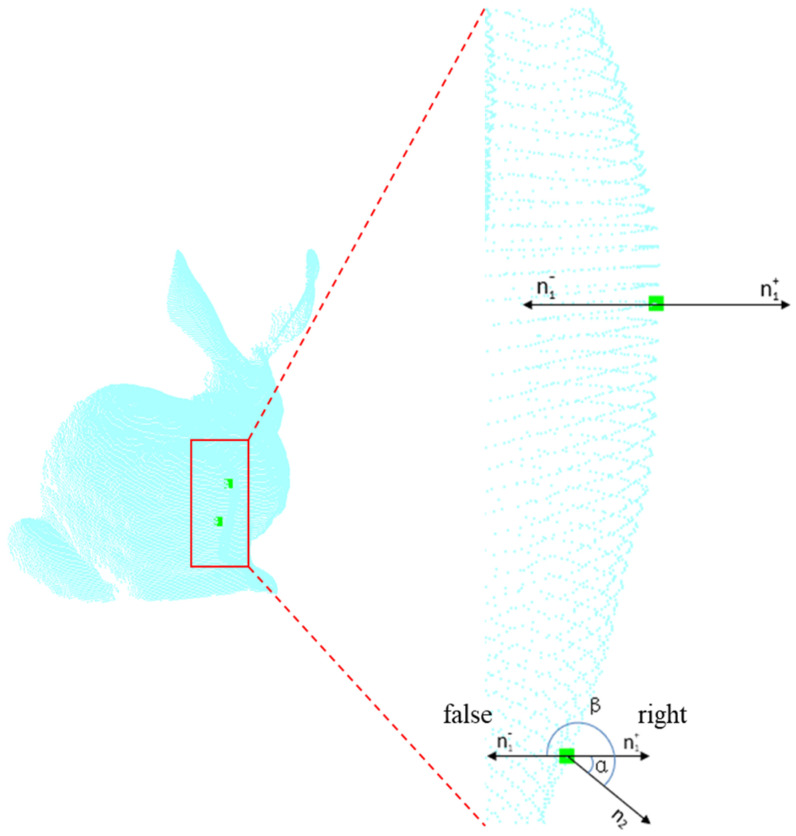
We use the two green points from the blue bunny object as a demonstration of the uncertainty of the normal direction. The uncertainty in normal directions arises when, for example, the direction $n_{1}^{+}$ is the correct normal direction, but $n_{1}^{-}$ is mistakenly chosen as the normal direction. In this case, the normal angle increases from α to β , resulting in an incorrect estimation of the normal angle.

The normal constraint [34] is another type of constraint used in point cloud registration. It utilizes the normal vectors associated with the points in the point clouds. After obtaining the normal, the normal constraint compares the angles between the normal vectors of two corresponding points in the source and target point clouds. This is achieved by calculating the cosine values of the angles (using the dot product of the unit normal vectors). The degree of the normal constraint is quantified by computing the absolute difference between the angles, as shown in (4).

(4) $$S_{\mathrm{normal}}\;=\left|\mathrm{acos}\left(n_{1}^{s}\cdot n_{2}^{s}\right)-\mathrm{acos}\left(n_{1}^{t}\cdot n_{2}^{t}\right)\right|$$

where n is the normal vector of point p . Both $n_{1}$ and $n_{2}$ are treated as unit normal vectors. So when the dot product is performed on these vectors, the resulting value is the cosine of the angle between the two vectors. The normal vectors are important features that describe the geometric properties of surfaces. The different normal angles between the source and target point clouds reflect the degree of disparity in surface curvature. Compared to the rigidity constraint, the normal constraint exhibits better robustness when dealing with noise and local variations. Even in the presence of noise or local non-rigid deformations, the normal constraint can still provide useful constraint information. However, the normal constraint only considers the normal angles between local correspondences, and it may not accurately capture the geometric information for cases involving large-scale shape variations or complex geometric structures. Moreover, the uncertainty in normal directions should also be taken into account. Selecting inconsistent normal directions as references can lead to erroneous angle calculations, resulting in misleading constraint results, as shown in Figure 4.

**Figure 5:**
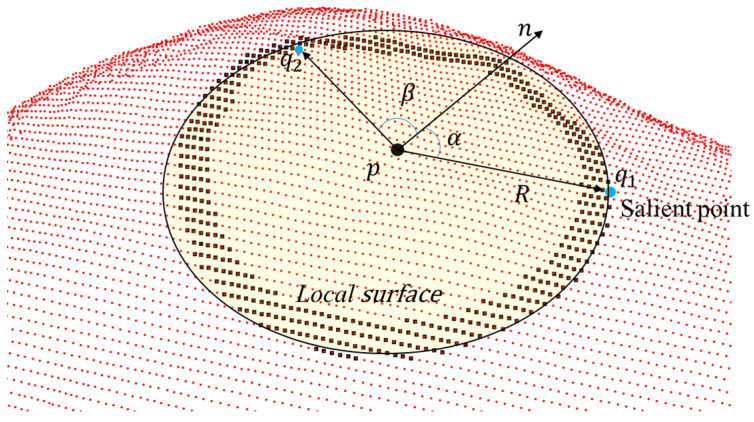
In the computation of salient points, for example, if the absolute value of vector $pq_{1}$ is greater than that of $pq_{2}$ , and the direction of vector $pq_{1}$ is more similar to the normal vector n compared to $pq_{2}$ ( cosα>cosβ ), we consider $q_{1}$ as the salient point. The black dots represent the points on the boundary region, and the blue dot represent the point that qualify as salient point on the boundary region.

The distance of the salient point (DSP) constraint [12] is derived based on the difference in distance between the center point p and its corresponding salient point $q^{*}$ . The salient point is selected from the boundary region of the local surface, satisfying two conditions: firstly, within this boundary region, the vector $pq^{*}$ has the longest length; secondly, the vector $pq^{*}$ has the most similar direction to the normal direction of centroid p . These conditions make the point $q^{*}$ exhibit saliency characteristics. An illustration of salient points is shown in Figure 5.

As shown in (5), $q^{*}$ represents a point on the boundary region that satisfies two conditions: the vector pq has the maximum length among all points in the boundary region, and its direction is consistent with the normal vector n of point p within a local surface with a radius of R . These imply that pq aligns to some extent with the tangent plane of the local surface. In this case, pq can be considered as a point on the surface with the maximum saliency.

(5) $$q^{⋆}=\underset{q}{\arg\max}|qp\cdot n|$$

The radius R of the local surface is determined by considering a neighborhood of points around p as shown in (6). Specifically, we select the $\left\lceil \frac{1}{pr}\right\rceil$ points $p_{nn_{i}}$ in the neighbor of p , denoted as k points. By using the point cloud resolution as a measure of the number of neighboring points, we ensure that the support radius is consistent with the sampling density of the point cloud. This choice of support radius allows for a better representation of the local surface’s geometric characteristics.

(6) $$R=\frac{1}{k}\left(\sum_{i=1}^{k}\left\|p-p_{{\mathrm{nn}}_{i}}\right\|\right)$$

After computing the salient points for each point, we apply a similar approach to the rigidity constraint by comparing the distance difference between $q_{1}^{*}$ and $q_{2}^{*}$ , as shown in (7).

(7) $$S_{\mathrm{dsp}}=\left|\left\|\left(q_{1}^{⋆s}-p_{2}^{s}p_{1}^{s}\right)-q_{2}^{⋆s}\right\|-\left\|\left(q_{1}^{⋆t}-p_{2}^{t}p_{1}^{t}\right)-q_{2}^{⋆t}\right\|\right|$$

**Figure 6:**
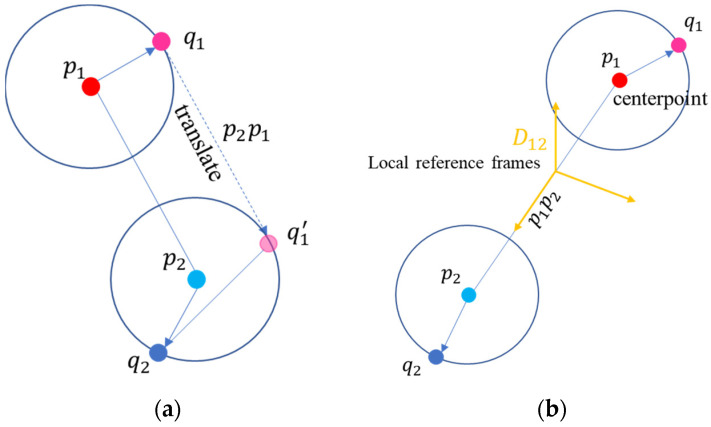
The red and blue points in the figure represent two source points from a grouping correspondences. The pink and dark blue points correspond to their salient points. Illustration of Salient points’ distance constraint (**a**); generating the transformation matrix with two correspondences (**b**) based on the centroid p an d the salient point q to establish a local reference frame (also demonstrated as the rotation pose in the transformation matrix).

However, in addition to this, we also translate the salient points by a vector $p_{2}p_{1}$ . By aligning the salient points with the center of $p_{2}$ , we eliminate the effect of spatial distance variation between $p_{1}$ and $p_{2}$ . This allows for a more accurate comparison of local structural differences in the two correspondences, taking into account not only the overall rigid transformation, but also the local variations, as illustrated in Figure 6a.

Afterwards, we combine the rigidity constraint and the distance of salient points constraint. These two constraints are eventually combined as $S_{both}$ to compute the score of the correspondences $c_{1}$ and $c_{2}$ given by (8).

(8) $$s_{\mathrm{both}}\;\left(c_{1},c_{2}\right)=\exp\left(-\frac{S_{\mathrm{rigidity}}}{{\left(a\cdot \mathrm{pr}\right)}^{2}}-\frac{S_{\mathrm{dsp}}}{{\left(b\cdot \mathrm{pr}\right)}^{2}}\right)$$

To weight the scores of each constraint, we use an exponential function. This allows the rigidity constraint ( $S_{rigidity}$ ) and the DSP constraint ( $S_{dsp}$ ) to have a more significant impact on decreasing the overall score if their values are higher, thus favoring the exclusion of correspondences that do not satisfy the constraints. In this formulation, a∗pr and b∗pr serve as distance thresholds for $S_{rigidity}$ and $S_{dsp}$ , respectively. When the constraints of correspondences are below these thresholds, it indicates that the geometric differences between the two correspondences are within an acceptable range, and the score will be maintained at a relatively high level. By setting appropriate values for a and b , we can achieve a desirable distance threshold. Here, pr represents the point cloud resolution, and it is defined by the Formula (9).

(9) $$\mathrm{pr}=\frac{1}{\left|P\right|}\sum_{p\in P}\frac{1}{10}\left(\sum_{i=1}^{10}\left\|p-p_{{\mathrm{nn}}_{i}}\right\|\right)$$

We calculate the average distance of the 10 nearest neighbor points $p_{nn_{i}}$ around each point p in the point cloud P . Then we compute the average distance of all points in the cloud. After that, we select the top-ranked correspondence combinations and calculate the local reference frame for the two correspondences $c_{1}$ and $c_{2}$ , as shown in (10).

(10) $$D_{12}=\left[\frac{p_{1}p_{2}\times \left(p_{1}q_{1}^{*}+p_{2}q_{2}^{*}\right)}{\left\|p_{1}p_{2}\times \left(p_{1}q_{1}^{*}+p_{2}q_{2}^{*}\right)\right\|}\;\frac{p_{1}p_{2}}{\left\|p_{1}p_{2}\right\|}\;\frac{p_{1}p_{2}\times \left(p_{1}q_{1}^{*}+p_{2}q_{2}^{*}\right)\times p_{1}p_{2}}{\left\|p_{1}p_{2}\times \left(p_{1}q_{1}^{*}+p_{2}q_{2}^{*}\right)\times p_{1}p_{2}\right\|}\right]$$

Here, we establish the first axis of the local reference frame based on the plane formed by $p_{1}q_{1}^{*}+p_{2}q_{2}^{*}$ and $p_{1}p_{2}$ . $P_{1}q_{1}^{*}+p_{2}q_{2}^{*}$ to integrate the information of the two salient points. Additionally, $p_{1}p_{2}$ serves as the second axis. We then construct the third axis, which is perpendicular to the plane formed by the first two axes, using the $p_{1}p_{2}\times \left({p_{1}q}_{1}^{*}+p_{2}q_{2}^{*}\right)\times p_{1}p_{2}$ vector. These three vectors form an orthogonal reference coordinate system, as shown in Figure 6b.

Finally, we can calculate the rotation and translation matrix based on the formula (11) proposed by Quan, S., and J. Yang [12].

(11) $$H={\left[\begin{array}{cc} D_{12}^{t} & \frac{p_{1}^{t}+p_{2}^{t}}{2}\\ 0 & 1 \end{array}\right]}^{-1}\left[\begin{array}{cc} D_{12}^{s} & \frac{p_{1}^{s}+p_{2}^{s}}{2}\\ 0 & 1 \end{array}\right]$$

In the formula, $H_{t}=\left[\begin{array}{cc} D_{12}^{t} & \frac{p_{1}^{t}+p_{2}^{t}}{2}\\ 0 & 1 \end{array}\right]$ and $H_{s}=\left[\begin{array}{cc} D_{12}^{s} & \frac{p_{1}^{s}+p_{2}^{s}}{2}\\ 0 & 1 \end{array}\right]$ represent the matrices formed by the center positions and rotation poses of two points in the source and target point clouds, respectively. However, in the formula $H_{t}\times H=H_{s}$ , the transformation matrix is applied to the target point cloud, returning it to the original coordinate system. We need to modify the formula to $H\times H_{s}=H_{t}$ so that the transformation matrix acts on the source point cloud. The modified transformation matrix formula should be as follows (12):

(12) $$H=\left[\begin{array}{cc} D_{12}^{t} & \frac{p_{1}^{t}+p_{2}^{t}}{2}\\ 0 & 1 \end{array}\right]{\left[\begin{array}{cc} D_{12}^{s} & \frac{p_{1}^{s}+p_{2}^{s}}{2}\\ 0 & 1 \end{array}\right]}^{-1}.$$

### 2.3. Matrix Evaluation

**Figure 7:**
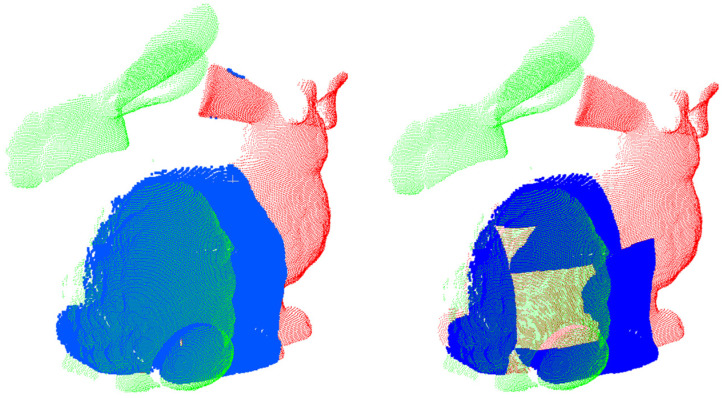
The green bunny model and the red bunny model represent unregistered point clouds. The blue areas represent their overlapping areas. In the left figure, the overlap ratio calculated using the kd-tree method is 60%, while in the right figure, the overlap ratio calculated using the octree method is 44%.

After generating the transformation matrix for the highly ranked correspondences, we need to evaluate the coarse registration results. We use two metrics to evaluate the results: overlap ratio and the number of inlier points. The overlap ratio can be calculated using two methods: KD-tree and octree. The KD-tree employs a nearest neighbor search algorithm to find the closest points to the query point within a given radius. On the other hand, the octree uses a voxelization approach to check for the existence of point samples within each voxel. Based on a comparative experiment between these two methods (as shown in Figure 7), we adopt the octree method to calculate the overlap ratio.

From the images, it can be observed that the octree method is able to more accurately identify the overlapping regions. Additionally, the octree method also demonstrates better search efficiency compared to the kd-tree method, which is beneficial for evaluating a large number of matrices and complex scenes. As for the specific steps of evaluating, firstly, we select the top-ranked registration results based on the overlap ratio threshold. Then, using the number of inlier points within the distance threshold to choose the best transformation matrix within these top-ranked results. We employ two metrics for evaluation for multiple reasons. Firstly, the overlap ratio considers the overall registration accuracy and reflects the alignment of the two point clouds on a global scale. Secondly, the number of inlier points takes into account the local quality of the registration results. Correct correspondences demonstrate consistency in geometric shape and topological structure within the local region. A higher number of inlier points signifies more precise and stable registration results within the overlapping region. Therefore, by considering both the overlap ratio and the number of inlier points, we can comprehensively evaluate the accuracy and completeness of the registration results.

Finally, we convert these main steps into Algorithm 1.
**Algorithm 1** Proposed registration method**Require**: Point clouds 
$p^{s}$
 and 
$p^{s}$
, and correspondence set 
C
; **Ensure**: Generate the best transformation matrix;1: Using TOLDIs descriptors and H3D keypoint detection to generate feature Histogram and using Equation (1) to generate initial correspondences; 2: Using Equation (2) to select the number of 
C

 
C=200;

3: Evaluate each correspondence based on the Equation (8)  in 
C
 using Equation (8);4: Ranking samples based on the compatibility score. Then we select out top 
N
 correspondences as candidates  
N=300
;5: **while** 
i<N
 **do**6: Compute transformation 
H
 based on 
${\left(c_{1},c_{2}\right)}_{i}$
 using Equation (11);7: Calculate inlier number 
$I_{i}$
 and overlap ratio 
$O_{i}$
 for each 
$H_{i}$
;8: 
i=i+1;

9: **end while**10: Setting threshold for overlap ratio 
$O_{T}=0.5;$
 Then we use 
$H_{T}$
 to store the 
$H_{i}$
 that fit 
$O_{T}$

11: **while** 
i<N
 **do**12:   **If**

$O_{i}>O_{T}$
 **then**13:    
$H_{T}\leftarrow H_{i}$
14:   **else** Re-enter the ovelap ratio until there is a point cloud exist15:   **end if**16:   
i=i+1;
17: **end while**18: Select the 
H
 from 
$H_{T}$
 with highest 
$I_{i}$
 as the optimal matrix.

## 3. Experiments and Discussion

This section focuses on verifying the accuracy of the point cloud coarse registration method proposed by this article. The entire experiment was conducted using the Point Cloud Library (PCL 1.12.1) with C++ programming language on a PC with an i7-9700 processor and 16 GB of RAM.

### 3.1. Experimental Setup

The experiment utilized various datasets, including Bunny, Dragon, and Armadillo from the Stanford dataset; kitchen and indoor scenes scanned by Princeton University; “Iqmulus & TerraMobilita Contest” [35] dataset is an urban environment in Paris, acquired by the French National Mapping Agency through mobile laser scanning (MLS); and the Taoist Zhenwu Temple in Rong County, Guangxi, collected by our team, as registration data (Table 1). These datasets exhibit different point cloud densities, overlap ratios, and application scenarios. By incorporating the diversity of these datasets, we can evaluate the practical applicability of the proposed method.

In terms of the evaluation criteria, the root mean square error (RMSE) [36] method is commonly used as a standard in various fields. We first calculated the distance error between corresponding points using the Formula (13).

(13) $$\epsilon_{p}\left(p^{s},p^{t}\right)=\left\|Rp^{s}+t-p^{t}\right\|$$

where R represents the rotation matrix and t represents the translation matrix. Additionally, $p^{s}$ and $p^{t}$ , respectively, represent the source point and the target point from the true correspondence. RMSE is defined as follows (14):

(14) $$\mathrm{RMSE}=\sum_{\left(p^{s},p^{\prime }\right)\in C_{\mathrm{gt}}}\frac{\epsilon_{p}\left(p^{s},p^{t}\right)}{\left|C_{\mathrm{gt}}\right|}.$$

$C_{gt}$ refers to the ground truth correspondences within the distance threshold. These correspondences are determined by comparing the distances between correspondences in correctly registered point cloud data. Specifically, the correspondences whose distances meet the distance threshold are considered as the ground truth correspondences. However, relying solely on the RMSE value does not effectively reflect the quality of point cloud registration. Some locally optimal matches can result in low RMSE values. The RMSE value becomes meaningful only when both point clouds are accurately aligned as a whole. Precision [37] was added to make the evaluation more comprehensive.

(15) $$\mathrm{Precision}=\frac{\left|C_{\mathrm{inlier}}^{\mathrm{corret}}\right|}{\left|C_{\mathrm{inlier}}\right|}$$

$C_{inlier}$ refers to the correspondences that satisfy the distance threshold after the registration process. Through multiple experiments, the distance threshold is typically set around 20*pr. Correspondences that meet this threshold ensure that the source and target point clouds are geometrically close. $C_{inlier}^{correct}$ specifically refers to the correct correspondences that are in $C_{inlier}$ and also meet the $C_{gt}$ standard. These correspondences are originally correct and remain accurate after registration process.

### 3.2. Analysis of Proposed Method

First, we investigate the influence of two key parameters on the registration accuracy of the method: the number of selected correspondences after NNSR selecting and the number of optimal transformation matrix. The experiment is conducted on the BUNNY090 and BUNNY180 datasets for registration. We use the TOLDI descriptor and Harris3D keypoint detection as the method for generating correspondences.

**Figure 8:**
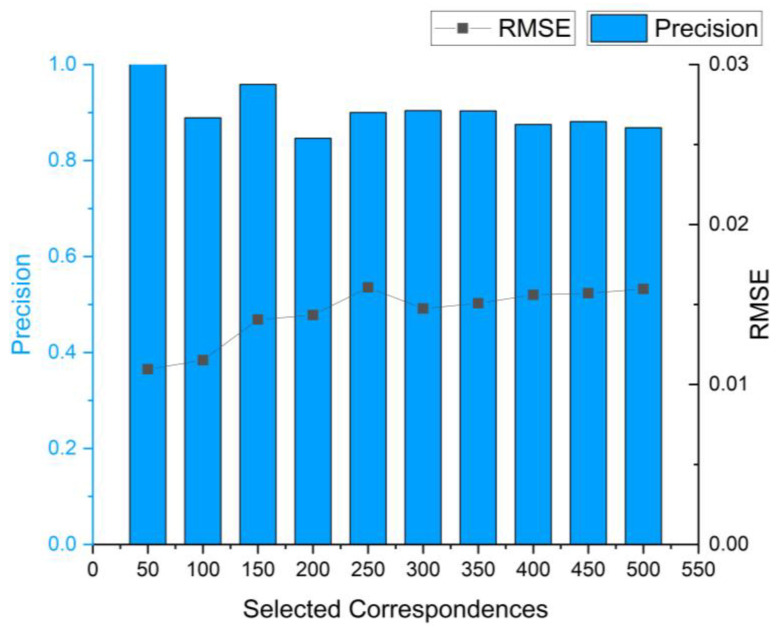
The impact of different numbers of correspondences selected by the NNSR method on registration accuracy.

According to the results shown in Figure 8, the precision and RMSE tend to stabilize when the NNSR method selects approximately 300 correspondences. After considering the trade-off between computational efficiency and registration accuracy, we choose 300 correspondences as the experimental parameter. The 300 correspondences perform well in terms of RMSE and precision, and provide a sufficient sample size for accuracy calculation, allowing for a more comprehensive evaluation of the practical effect of point cloud registration.

**Figure 9:**
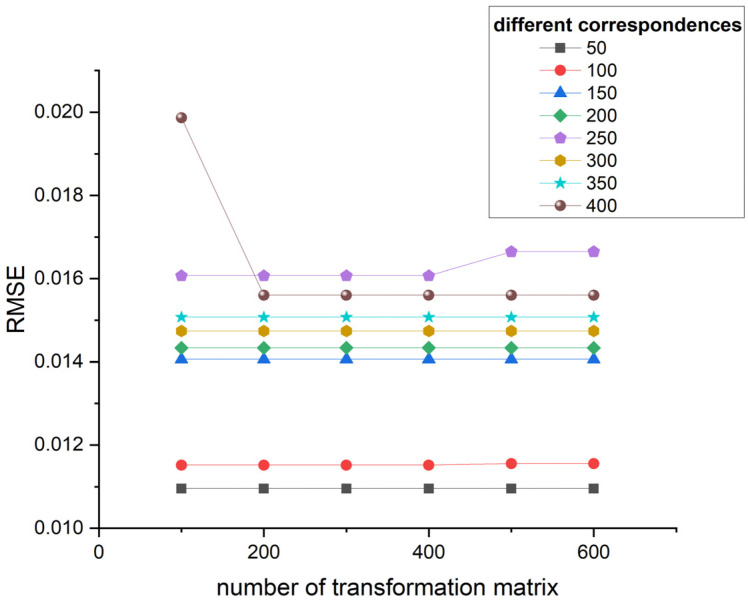
Registration results of different hypothetical transformation matrices corresponding to different correspondences.

Based on the results shown in Figure 9, different transformation matrices yield consistent registration results ranging from 100 to 600, except for 400 and 250 correspondences. Considering the number of provided transformation matrices for evaluation and computational efficiency, we choose 300 hypothesized transformation matrices as the optimal parameter. Having too many transformation matrices can increase the computation time for metrics such as overlap ratio and inlier count, while having too few may result in missing some high-quality transformation matrices.

**Figure 10:**
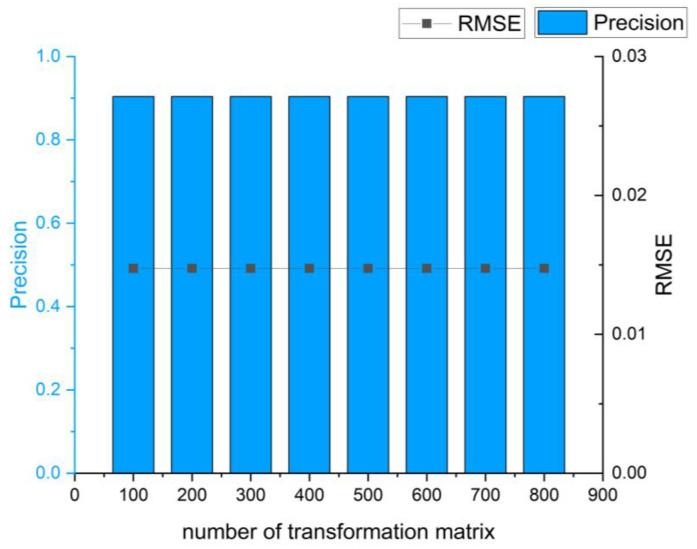
Influence of different hypothetical transformation matrices on registration accuracy under 300 correspondences.

Figure 10 illustrates the influence of different transformation matrices on RMSE and precision in the case of 300 correspondences. From the graph, it can be observed that the accuracy results remain consistent across 100 to 800 transformation matrices. This indicates the uniqueness of the optimal matrix within this number of correspondences.

In determining the settings for the distance threshold of the inliers and overlap ratio parameters, based on multiple experiments, we determined to set the distance threshold in the range of 15*pr to 20*pr. This range allows us to assess the proximity between point pairs. The overlap ratio is also a parameter used to evaluate registration accuracy. We utilize the pre-registration overlap ratio as a threshold, as shown in Table 1. This approach helps eliminate misalignments introduced during the registration process and visually showcases the improvements achieved in numbers after registration.

**Figure 11:**
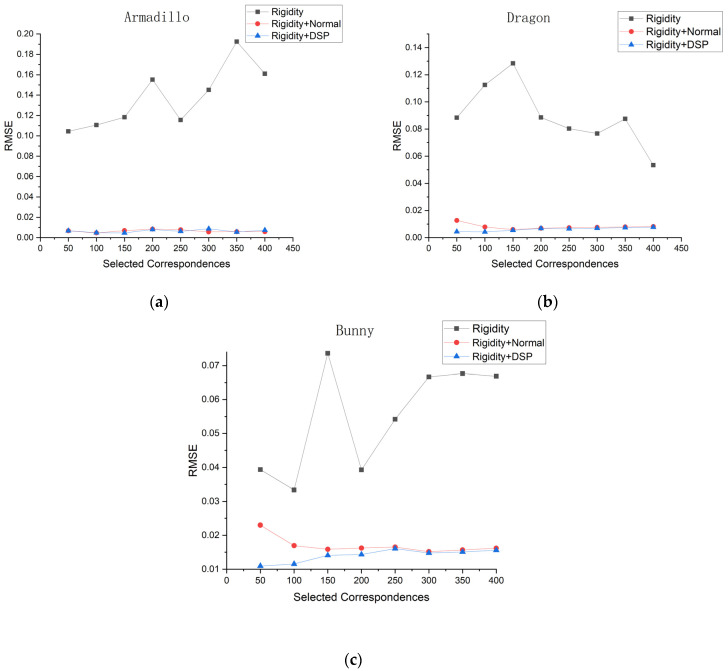
(**a**–**c**) respectively represents the registration accuracy of Armadillo, Dragon, and Bunny models respectively under different geometric constraints.

Subsequently, to validate the superiority of combining rigidity constraints with distance of salient point constraints, we conducted tests on three different approaches: rigidity constraint with distance of salient point constraint, rigidity constraint only, and rigidity constraint with normal constraint. We conducted tests on the RMSE values of different object under various geometric constraints (as shown in Figure 11). The registration results of the three different objects clearly indicate that the registration accuracy of the two geometric constraints is superior to using only a single rigidity constraint. By adding the normal constraint, we ensure the similarity in the normal direction of correspondences. Additionally, the salient point constraint utilizes local surface information and provides effective assistance in situations where the rigidity constraint may be ambiguous.

The RMSR value of the Rigidity + DSP constraint can be observed to be superior to the Rigidity + Normal constraint at 50 to 200 correspondences. Both constraint exhibit similar RMSE when the number of correspondences reaches 300. However, there is still an average difference of 0.0005 between them in the RMSE value, proving that the Rigidity + DSP constraint is still superior. As shown in , overall, the combination of the rigidity constraint with the distance of salient point constraint yields better registration results.

**Figure 12:**
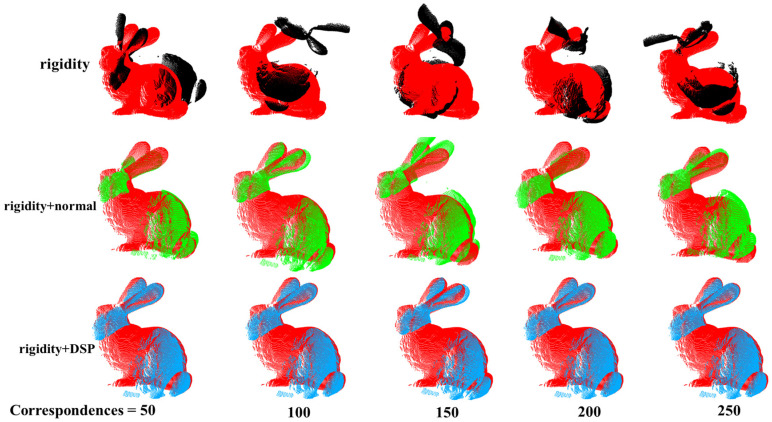
The red bunny represents the target point cloud. The black bunny represents registration under Rigidity constraints. Green one represents registration under the Rigidity + Normal constraint. The blue one represents registration under the constraints of Rigidity + DSP. Figure shows registration result under different geometric constraints.

In the case of the Bunny, the RMSR value of the Rigidity + DSP constraint can be observed to be superior to the Rigidity + Normal constraint at 50 to 200 correspondences as shown in Figure 12. Both constraints exhibit similar RMSE when the number of correspondences reaches 300. However, there is still an average difference of 0.0005 between them in the RMSE value, proving that the Rigidity + DSP constraint is still superior. The trends of RMSE value in the Dragon are similar to those of the Bunny, as shown in Figure 11b, while in the Armadillo(Figure 11c), both methods exhibit a nearly constant trend. Overall, the registration performance of Rigidity + DSP is superior to Rigidity + Normal when the number of correspondences is small.

**Figure 13:**
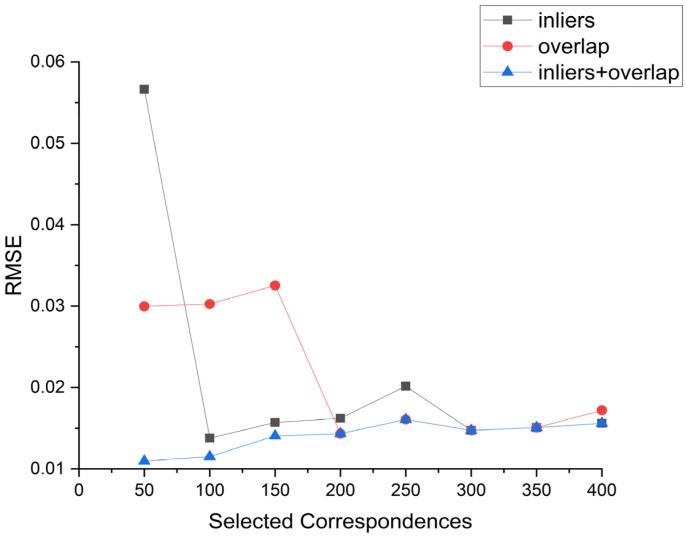
Registration accuracy under different evaluation standards.

**Figure 14:**
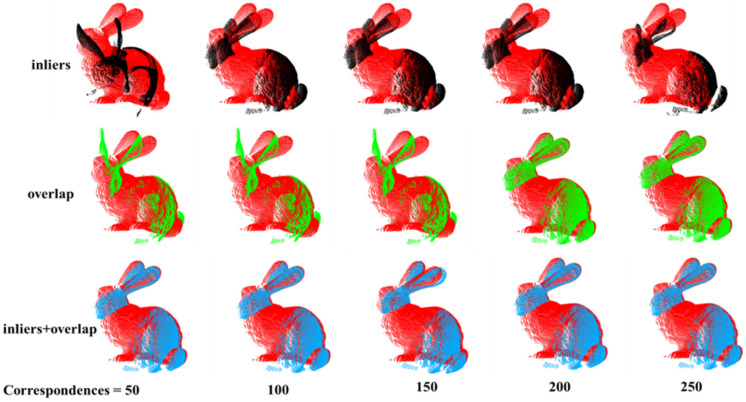
The black bunny represents registration under inliers evaluation criteria. Green one represents registration under the overlap evaluation criteria. The blue one represents registration under the inliers + overlap evaluation criteria. Figure shows registration result under different evaluation criteria.

Then, we compared the registration results using different evaluation methods. We conducted tests on the Bunny model and compared the performance of these methods, as shown in Figure 13 and Table 2. These evaluation methods include using the maximum number of inliers and the highest overlap ratio for assessment. We can observe that the method combining inliers and overlap ratio consistently produces the best registration matrix overall. The method based on overlap ratio reaches a similar performance to the inliers and overlap ratio method after generating approximately 200 correspondences. On the other hand, the method based on the maximum number of inliers shows more fluctuations in its registration results and only catches up with the inliers + overlap ratio method when the number of correspondences exceeds 300. In conclusion, among these three evaluation methods, combining the two criteria leads to better registration results. The registration results of Bunny using different evaluation methods are shown in Figure 14. We selected a range of 50 to 250 correspondences for comparison. It can be observed that, overall, the method combining inliers and overlap consistently achieves good registration results at any number of point pairs.

**Figure 15:**
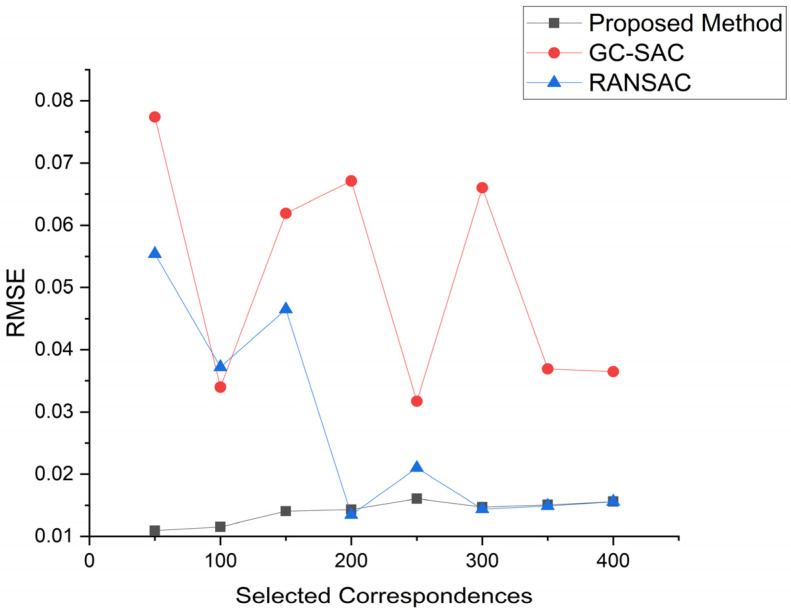
Registration accuracy under different transformation matrix estimators.

**Figure 16:**
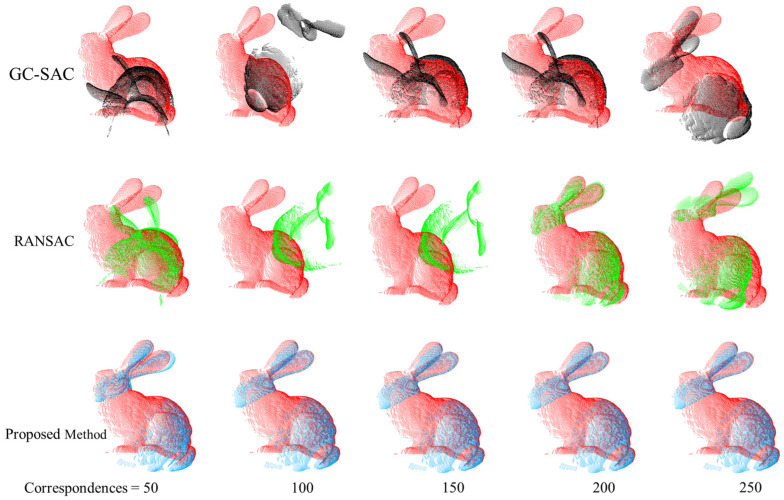
The black bunny represents registration under GC-SAC estimator. Green one represents registration under the RANSAC estimator. The blue one represents registration under our estimator. Figure shows registration result under different transformation matrix estimators.

Finally, we conducted tests on different transformation matrix calculation methods, and the results are shown in Figure 15 and Table 3. Firstly, we compared the proposed method with the GC-SAC method, which is using a different matrix calculation formula. Additionally, it can be seen that the proposed method outperforms the GC-SAC method in terms of registration accuracy. Then, we adopted the RANSAC method to remove the outlier proposed by X. L. [38], and combined it with singular value decomposition (SVD) to calculate the optimal transformation matrix. The results show that in the registration results with 50 to 250 selected correspondences, the proposed method is superior to the RANSAC method overall. In the range of 300 to 400 selected correspondences, the proposed method has a slightly higher average RMSE value compared to the RANSAC method, with a difference of 0.00018. However, the proposed registration method still achieves good results overall, as shown in Figure 16.

**Figure 17:**
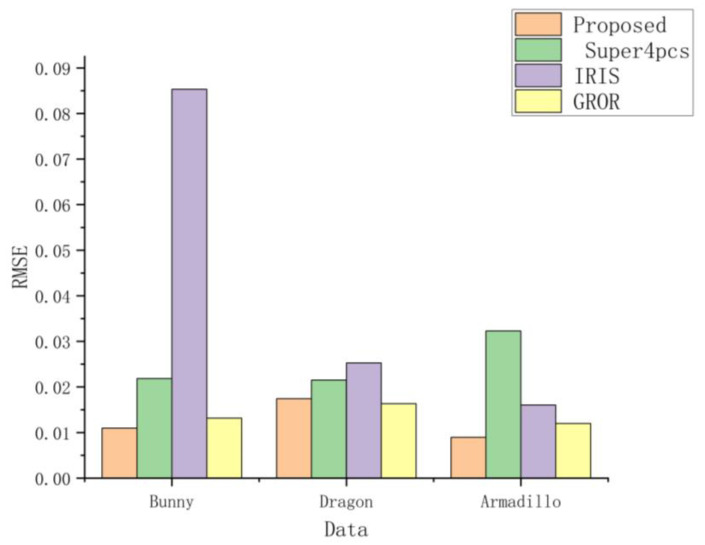
Registration accuracy under different registration methods.

**Figure 18:**
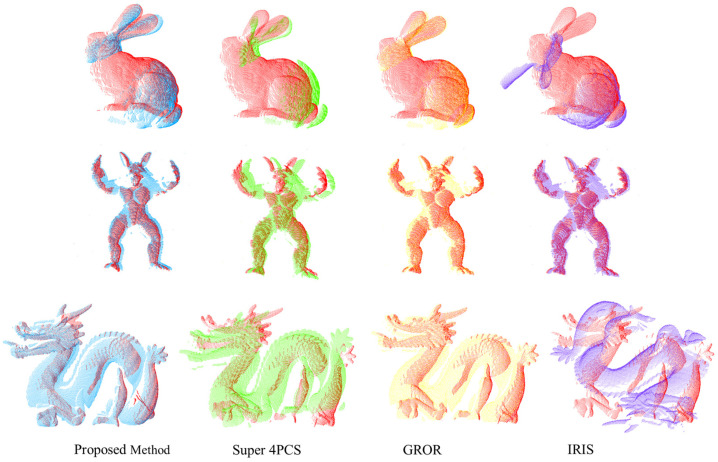
Blue, green, yellow and purple respectively represents our proposed method, Super4PCS, GROR and IRIS. Figure shows Registration results under different registration methods.

After confirming the entire registration process, we compared the entire registration process of the proposed method, along with some of the feature-matching registration methods such as IRIS [20], GROR [21], and Super 4PCS [39]. The results are present in Figure 17 and Figure 18. We can observe that in the comparison of three different point cloud objects, the proposed method outperforms the other three methods in terms of RMSE value and actual registration performance. Additionally, the proposed method is similar to Super 4PCS, which also utilizes geometric constraints such as limiting distance range and angle to find the optimal identical four-point matches. However, the proposed method has a lower computational time compared to Super4PCS.

### 3.3. Proposed Method in Practice

**Figure 19:**
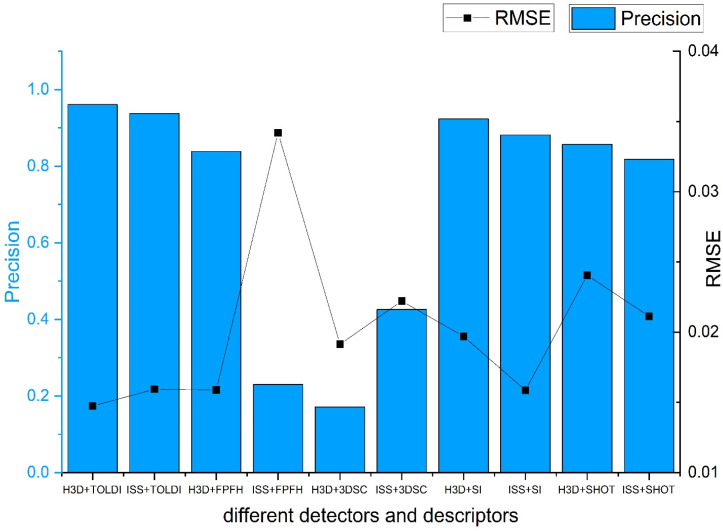
Different combinations of descriptors and keypoint detection methods on registration accuracy.

**Figure 20:**
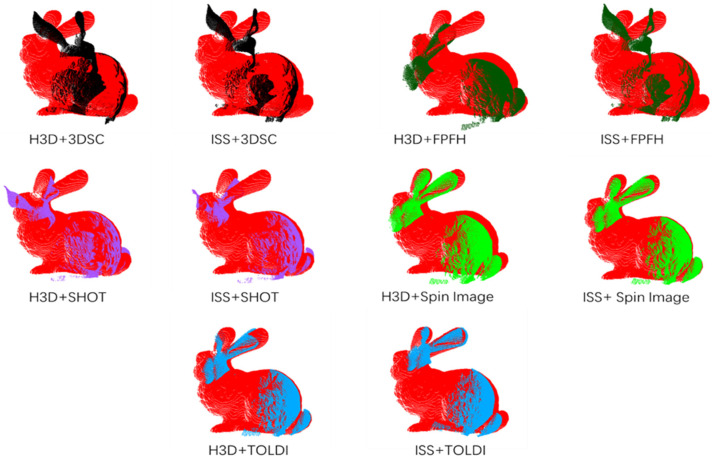
The different colors represent the registration results under different descriptors. Black, dark green, purple, green and blue respectively represents 3DSC, FPFH, SHOT, Spin Image and TOLDI descriptor. Figure shows different combinations of descriptors and keypoint detection methods on registration results.

Based on the BUNNY090 and BUNNY180 datasets, we evaluated the registration performance of the method using different descriptors and keypoint detection methods. The keypoint detection methods included Harris 3D (H3D) and intrinsic shape signatures (ISS). The descriptors included fast point feature histograms (FPFH), 3D shape context descriptor (3DSC), signature of histograms of orientations (SHOT), spin image (SI), and TOLDIs. We assessed the accuracy of the registered point clouds using different descriptors and keypoint detection methods (Figure 19), as well as the visual quality of the registration results (Figure 20). Detailed results after registration are shown in Table 4.

Observing Figure 19 and Figure 20, it can be noted that under the constraints of rigidity and salient points’ distance, most of the methods achieve good actual registration results. The RMSE values are maintained around 0.01 to 0.02, and the precision also exceeds 80%. However, the 3DSC descriptor, in combination with both keypoint detection methods, and the FPFH descriptor with the ISS keypoint detection method, did not meet the expected standards in terms of actual registration results.

In the comparison between the combinations of H3D and ISS methods with different descriptors, it can be observed that the registration results with H3D keypoint detection are slightly better than those with ISS keypoint detection. This is because our experimental dataset, Bunny, is more sensitive to corner point features such as the ears and nose of the rabbit, and the Harris 3D method is more suitable for capturing these corner point features. On the other hand, the ISS algorithm focuses on capturing more comprehensive keypoint information of the model, including curvature and normal changes. From the experimental results, it can be concluded that the corner point features of the rabbit have more distinctive characteristics compared to its ISS keypoints.

In terms of descriptors, TOLDI utilizes projections in three orthogonal directions, allowing it to capture local shape features of the point cloud data in different directions. This enables TOLDI to capture more detailed and local structural information in multiple dimensions. This is the reason why TOLDI performs well among all the descriptor methods. The spin image descriptor achieves the second best performance. Since the BUNNY090 and BUNNY180 datasets are obtained from different viewpoints, the spin image descriptor, which calculates rotational projection histograms on the point cloud, can describe local geometric features and counteract noise and inconsistencies between local point clouds through rotational invariance. As a result, it demonstrates good matching performance on point cloud data acquired by rotating at different angles.

The SHOT descriptor has relatively high dimensions, typically around 352 dimensions, while the FPFH descriptor has relatively low dimensions, typically around 33 dimensions. Therefore, the registration results achieved by the SHOT descriptor are superior to the FPFH descriptor. The 3DSC descriptor encodes the geometric relationships between points on a spherical surface and their neighboring points, providing a more comprehensive representation of the overall shape of the point cloud. On the other hand, the FPFH descriptor focuses on the relative angular changes between the neighboring points’ normal changes and is suitable for surfaces with significant normal variations or objects with edge features. For the Bunny model, which emphasizes local features, the FPFH descriptor slightly outperforms the 3DSC descriptor.

**Figure 21:**
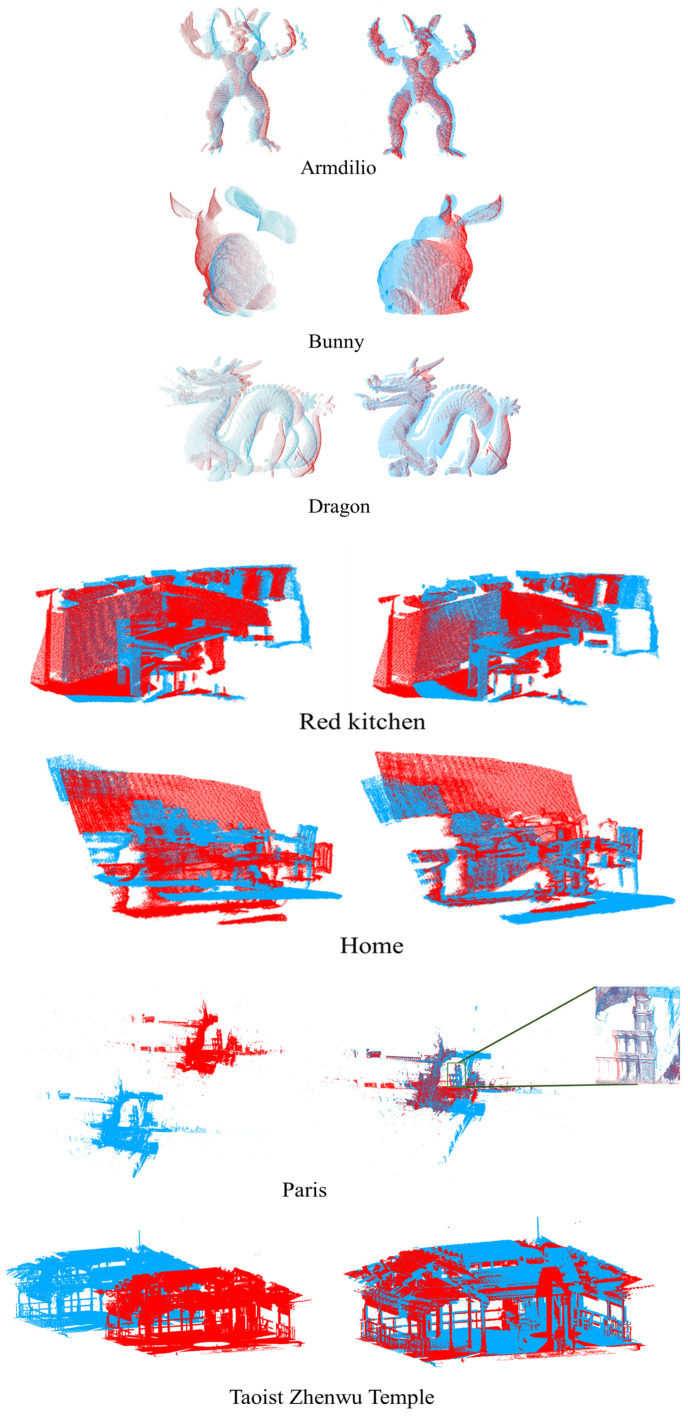
The coarse registration results of each data based on geometric constraints and comprehensive evaluation, the left is before registration and the right is after registration. Blue represents source point cloud and red represent target point cloud.

To test the robustness of proposed registration method on different data types, we conducted experiments on seven datasets: Bunny, Dragon, Armadillo, RedKitchen, Home, Paris, and the Taoist Zhenwu Temple. The Taoist Zhenwu Temple dataset was acquired using the Riegl VZ-1000 3D laser scanner in Rong County, Yulin City, Guangxi, China. It can be observed from Table 5 that the precision of the registrations using the geometric constraints and comprehensive evaluation method remain above 80%. The RMSE values are also below 15pr. Even for complex indoor scenes such as the Taoist Zhenwu Temple, the RMSE is around 5pr. The actual registration results for each dataset are shown in Figure 21.

**Figure 22:**
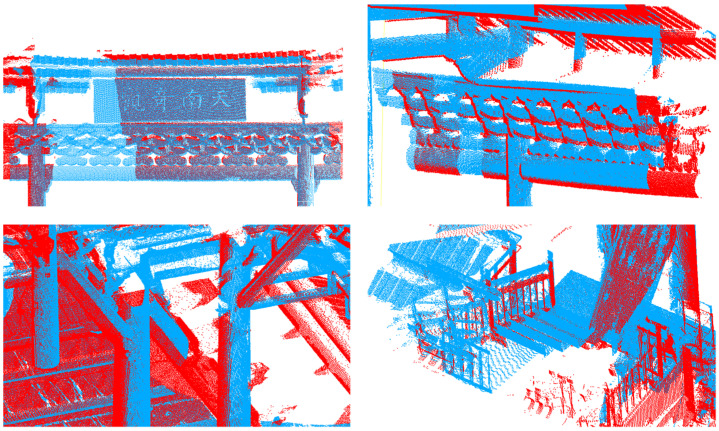
The upper right image shows the nameplate(southern wonder) of the building. The upper left image depicts decorative beams. The lower right image displays beams and pillars within the building. The lower left image shows the junction at the staircase. In this figure, red and blue represent the Zhenwu Temple scanned from different angles.

Figure 22 displays the local details of the registration for the Taoist Zhenwu Temple. Even for complex historical architectural structures and with voxel filtering applied to the raw data, the proposed method can achieve structural registration. This verifies that the registration method is capable of providing high-quality registration results for complex structures and multi-scale data.

## 4. Discussion

Our experimental observations distinctly illustrate the superiority of point-to-point correspondence-based registration methods compared with others. The approach that employs a combination of rigidity constraints and distance of salient point constraints yields a more accurate set of correspondences when contrasted to other geometric constraints. The key advantage of integrating rigidity and DSP constraints is that DSP takes into account local geometric details, effectively resolving ambiguities introduced by normal constraints and rigidity constraints.

When it comes to generating transformation matrices and selecting the best transformation, through experiments, it was shown that the proposed method for creating transformation matrices, based on pairs of points from both the source and target point clouds, outperforms conventional techniques such as RANSAC and GC-SAC. Because of the high-quality correspondences, the proposed method achieves improved efficiency and accuracy in registration results compared to other estimator methods. Additionally, the proposed evaluation method, which combines inlier points and overlap ratio, provides a comprehensive assessment of both local and global qualities of the registration results. This innovative evaluation approach outshines other methods and contributes to a more comprehensive understanding of registration outcomes. Once the registration process was set, we compared our proposed method with three existing feature-based registration methods. The results indicate that our method also outperformed similar registration methods in terms of accuracy. The experiment also proves the robustness of the proposed coarse registration method in real-world applications, as it performs well in terms of precision and practical effectiveness when tested with different types, overlap ratios, and point cloud densities of data.

However, it is important to acknowledge the limitations inherent in our experimental approach. It should be noted that the results, those concerning different estimators, and evaluation techniques, were derived from the Bunny model. To ascertain the method’s efficacy across varied datasets, future research should encompass more extensive experiments. Furthermore, in the comparative experiments involving recent methods, we encountered several challenges due to time constraints. Specifically, we encountered the following issues:

(1) Due to our incomplete understanding of the underlying principles of the other three methods, achieving optimal registration results was challenging.
(2) The selection of comparable methods for our study was constrained by a limited pool of options. Additionally, some of the chosen methods may not accurately represent the latest advancements in registration techniques. This aspect limited the breadth and accuracy of our comparative analysis.
(3) By not integrating other established point cloud registration evaluation metrics, the comprehensiveness of our results was compromised, and as a result, the overall persuasiveness of our findings was diminished.

## 5. Conclusions

In this paper, we propose a coarse registration method based on local geometric feature constraints, combined with a comprehensive evaluation of inliers and overlap ratio. The main steps of this method include correspondences filtering, transformation matrix computation, and evaluation of matrix. First, we combine the constraints of the salient points’ distance and rigidity to select high-quality correspondences. Then, based on the centroids of the correspondences and their reference frames, we compute the transformation matrix for each correspondence. Finally, using the evaluation metrics of inliers and overlap ratio, we select the best registration matrix. We compare different descriptors and feature point detection methods to choose the one with the highest registration accuracy as our experimental approach. Additionally, we compare the effects of different geometric constraints on the experimental results and demonstrate that the constraints of salient points’ distance and rigidity yield better results. By comparing single evaluation criteria, we show that the overall registration results are improved when both evaluation metrics are considered. Finally, we test our registration method on different types of datasets to demonstrate its robustness and accuracy. In future work, the repetitive evaluation process still significantly consumes time. There is still room for us to optimize the code in order to improve computational efficiency. Additionally, the current method only considers the geometric aspects (XYZ) of the point cloud data. We aim to incorporate additional parameters, such as RGB and intensity, in the subsequent registration experiments to enhance the coarse registration process. These parameters can provide valuable information and improve the accuracy of the coarse registration by considering not only geometric features, but also color and intensity characteristics.

## Figures and Tables

**Table 1:** Experimental data.

No.	Data	Source Points	Target Points	Overlap Ratio	Resolution	Scenario
1	Bunny	30,379	40,251	0.60304	0.001	Object
2	Dragon	41,841	22,092	0.36715	0.00099	Object
3	Armadillo	26,941	25,570	0.81912	0.001	Object
4	redKitchen	258,342	268,977	0.47331	0.0081	Indoor
5	Home	425,577	373,295	0.74085	0.008	Indoor
6	Paris	372,620	204,128	0.85941	0.73	Outdoor
7	Zhenwu Temple	2,273,238	2146,665	0.84996	0.018	Indoor

**Table 2:** RMSE result of three evaluation methods.

Selected Correspondences	Inliers	Overlap Ratio	Inliers + Overlap
50	0.05663	0.02998	0.01096
100	0.0157	0.03027	0.01152
150	0.01622	0.03254	0.01406
200	0.02018	0.01433	0.01433
250	0.01474	0.01607	0.01607
300	0.0667	0.01474	0.01474
350	0.01507	0.01507	0.01507
400	0.0156	0.0172	0.0156

**Table 3:** RMSE result of three transformation matrix estimators.

Selected Correspondences	Proposed	GC-SAC	RANSAC
50	0.01096	0.07739	0.05538
100	0.01152	0.03398	0.0372
150	0.01406	0.06191	0.04648
200	0.01433	0.06711	0.01347
250	0.01607	0.03172	0.02102
300	0.01474	0.06604	0.0144
350	0.01507	0.03694	0.0149
400	0.0156	0.03648	0.01559

**Table 4:** Different descriptors and keypoint detections combined with the proposed method.

Method	RMSE	Precision
TOLDI + H3D	0.014741	0.96
TOLDI + iss	0.01594	0.94
Fpfh + H3D	0.01590	0.84
Fpfh + iss	0.03420	0.23
SI + H3D	0.0197	0.92
SI + iss	0.01586	0.88
3DSC + H3D	0.01914	0.17
3DSC + iss	0.02222	0.43
SHOT + H3D	0.02405	0.8
SHOT + ISS	0.02113	0.82

**Table 5:** Registration results for different types of data.

Data	RMSE	Precision
Bunny	0.01474	0.90385
Dragon	0.01742	0.8932
Armadillo	0.00894	1
redKitchen	0.02249	0.84906
Home	0.04516	0.827
Paris	0.52034	0.948
Taoist Zhenwu Temple	0.135652251	0.850746269

## Data Availability

Not applicable.

## Abbreviations

The following abbreviations are used in this manuscript:

RMSE	Root Mean Square Error
ICP	Iterative Closest Point
GC-SAC	compatibility-guided sampling consensu
RoPS	Rotation Projection Statistics
3DHV	3D Hough Voting
PCV	Progressive Consistency Voting
ISS	intrinsic shape signatures ISS
3DSC	3D shape context descriptor
GROR	graph reliability outlier removal
RANSAC	Random Sample Consensus
OASC	Optimized Sample Consensus
H3D	Harris 3D
SI	Spin Image
LRF	Local Reference Frame
FPFH	Point Feature Histogram
SHOT	Signature of Histograms of Orientations
NNSR	Nearest Neighbor Similarity Ratio
PCRMLP	point cloud registration with multilayer perceptrons
DBSCAN	density-based spatial clustering of applications with noise
TODLI	triple orthogonal localdepth images
DSP	distance of salient point
PCL	Point Cloud Library
MLS	Mobile Laser Scanning
SVD	Singular Value Decomposition
