# Correction: Kang et al. Point Cloud Registration Method Based on Geometric Constraint and Transformation Evaluation. *Sensors* 2024, *24*, 1853

**DOI:** 10.3390/s26041241

**Published:** 2026-02-14

**Authors:** Chuanli Kang, Chongming Geng, Zitao Lin, Sai Zhang, Siyao Zhang, Shiwei Wang

**Affiliations:** 1College of Geomatics and Geoinformation, Guilin University of Technology, Guilin 541004, China; 2014012@glut.edu.cn (C.K.); 2120211878@glut.edu.cn (Z.L.); 1020211817@glut.edu.cn (S.Z.); 2120211915@glut.edu.cn (S.Z.); 2120222025@glut.edu.cn (S.W.); 2Key Laboratory of Spatial Information and Geomatics, Guilin University of Technology, Guilin 541004, China

## Text Correction

In the original publication [1], there was an error. A correction has been made to Section 1, paragraph 3. The content “Abdullah Lakhan et al. [12] proposed a DAPWTS algorithm framework, which utilizes a secure minimum cut algorithm to partition applications between local nodes and edge nodes. After the application partitioning, an optimal search is performed using a node search algorithm, which also optimizes the structure of point cloud data.” was removed.

## References

Reference [12] was removed. With this correction, the order of some references has been adjusted accordingly.

The authors state that the scientific conclusions are unaffected. This correction was approved by the Academic Editor. The original publication has also been updated.
